# Foxp3^+^ T Regulatory Cells: Still Many Unanswered Questions—A Perspective After 20 Years of Study

**DOI:** 10.3389/fimmu.2018.01048

**Published:** 2018-05-15

**Authors:** Ethan M. Shevach

**Affiliations:** Laboratory of Immune System Biology, National Institute of Allergy and Infectious Diseases, Bethesda, MD, United States

**Keywords:** T regulatory cells, tolerance mechanisms, Foxp3, suppression mechanisms, autoimmune diseases

## Abstract

T regulatory (Treg) cells were discovered more than 20 years ago and have remained a topic of intense investigation by immunologists. The initial doubts about their existence were dissipated by the discovery in 2003 of the lineage specific transcription factor Foxp3. In this article, I will discuss some of the questions that I believe still need to be answered before we will be able to fully apply Treg therapy to the clinic. The major issue that remains to be resolved is how they mediate their suppressive functions. In order to correct defective suppression in autoimmune disease (assuming it is a causative factor) or to augment suppression in graft versus host disease or during organ transplantation, we still need to fully understand the biochemical nature of suppressor mechanisms. Similarly, in cancer, it is now widely accepted that reversal of Treg suppression would be highly desirable, yet which of the many purported pathways of suppression are operative in different tumors in different anatomic sites. Many of the concepts we have developed are based on *in vitro* studies, and it remains unclear if these concepts can readily be applied to Treg function *in vivo*. Our lack of a specific cell surface marker that readily allows us to identify and target Treg *in vivo*, particularly in man, remains a major stumbling block. Finally, I will review in some detail controversies regarding the origin of Treg, thymus versus periphery, and attempts to reverse Treg suppression by targeting antigens on their cell surface, particularly members of the TNF receptor superfamily. Hopefully, these areas of controversy will be resolved by in depth studies over the next few years and manipulation of Treg function will be placed on a more solid experimental footing.

## William E. Paul: In Memoriam

I first met Bill Paul in 1971 at an extremely low point in my career. I was looking for a new supervisor for my postdoctoral training as I had just spent about 18 months working in a lab where I had accomplished absolutely nothing. Bill had just been appointed Chief of the Laboratory of Immunology, was quite understanding of my situation, and advised me to speak with Ira Green about potential opportunities in his lab. Ira took me on as postdoc and pointed me in the right direction. Bill also assumed a co-supervisory role particularly on projects that he and Ira had studied together for many years dealing with the function of immune response genes. I thrived in this environment and after only two full years as a postdoc was offered a tenured position in the Laboratory of Immunology where I have remained for the past 45 years. My lab and Bill’s lab were immediately adjacent to each other on the 11th floor of the Clinical Center and we had numerous interactions on a daily basis. For over 20 years we had joint data and journal clubs for our groups every Wednesday and Friday morning. One fringe benefit of these discussions was that my postdoctoral fellows benefited from Bill’s wisdom and criticism. His comments were always delivered in a gentle fashion often pointing out major areas of deficiency or steps in the wrong direction. The fellows always accepted them and never felt threatened as they were always perceived as constructive. I am not certain my comments on his fellow’s presentations were always similarly perceived! When we began our studies on T regulatory cells even I was somewhat leery as to how Bill would react to our attempts to redefine T suppression after its death in the 1980s. Bill was actually quite receptive of our approach and continued to encourage me to continue even after I received a negative review from our advisory committee. He was particularly proud to announce to the committee that I received the William Coley Award in 2004 for our studies on regulatory T cells in spite of their negative comments.

## Introduction

In 2002, I wrote a review entitled “CD4^+^CD25^+^ Suppressor T Cells: More Questions Than Answers (1).” Foxp3 had yet to be discovered as the marker for this lineage and the term “Regulatory” rather than “Suppressor,” had not yet become the convention. Over the past 15 years, this field has seen tremendous growth and the therapeutic manipulation of T regulatory (Treg) function has reached the clinic. Certain aspects of the field that have received great attention and many of the questions I posed in 2002 have been answered. However, some questions remain unanswered and our lack of knowledge of these aspects of the field in my view has clearly hindered progress in the clinical application of Treg either to boost their function in autoimmunity or disable their function in malignancy. In this review, I will focus on several questions that I believe remain unanswered.

## Assays of Treg Function *In Vitro*

My group (2) and the Sakaguchi group (3) described the first assays for the measurement of the suppressor function of CD4^+^CD25^+^ T cells *in vitro*. Although this type of assay was rapidly adopted by almost all investigators in the field, a number of issues have emerged that render interpretation of the results of these experiments problematic. In general, these assays involve the measurement of the proliferation of mouse non-Treg cells (either CD4^+^ or CD8^+^) triggered by TCR signaling in the presence of a titration of highly purified Treg cells. In the original studies, soluble anti-CD3 stimulation was used to trigger the TCR and the assay was always performed in the presence of accessory cells (T-depleted spleen cells, or more recently dendritic cells) that were needed to cross-link the anti-CD3 antibody and provide co-stimulatory signals. The addition of anti-CD28 was not recommended, as it was more difficult to achieve significant suppression with greater levels of TCR stimulation. The basis for this recommendation was the observation that Treg primarily inhibited proliferation by blocking IL-2 production by the responder population and anti-CD28 enhances IL-2 production by prolonging IL-2 mRNA half-life. The initial studies attempting to adapt this assay for use with human Treg frequently incorporated anti-CD28 co-stimulation to achieve significant levels of stimulation. While suppression was observed under these culture conditions, higher numbers of Tregs were required to achieve significant suppression and ratios of 1:1 (Treg:responder) were frequently employed. However, assay conditions very similar to those used in the mouse can be used with human cells (4). Significant levels of stimulation in the absence of anti-CD28 with the most commonly used anti-CD3 antibodies (OKT3 and UCHT1) can readily be achieved when a population of HLA-DR^+^ non-T cells are used as an accessory cell population.

A number of investigators questioned the use of the soluble anti-CD3 and accessory cell approach and claimed that the use of a defined number of anti-CD3 coated or anti-CD3 and anti-CD28 coated beads was a much more precise method for stimulating T cell activation. Although Tregs are capable of inhibiting responses induced by this activation protocol, suppression again almost always required 1:1 or at best 1:2 ratios of Treg to responder cells and no suppression was frequently seen at lower ratios of Treg to responder cells. A number of questions can be raised about the use of antibody bound to beads or anti-CD3 coated plates. T cell stimulation by antibody coupled to solid surfaces may result in a qualitatively distinct signal from stimulation induced by antigen presented on professional APC or even soluble anti-CD3 stimulation in the presence of APC. In our initial studies in the mouse on Treg suppression *in vitro* (2), we found that it was exceedingly difficult to suppress T cell stimulation induced by plate bound anti-CD3. Furthermore, this resistance to suppression was not overcome by using lower concentrations of anti-CD3 to coat the plate. Our interpretation of this result was that fewer T cells were triggered to proliferate at lower concentration of plate bound antibody, but that every T cell that bound to the solid phase stimulus still received a potent signal which was resistant to Treg-mediated suppression. This question has yet to be resolved and the use of a two cell assays versus a three cell assay remains controversial.

The second issue raised by these experiments is the cellular target of Treg-mediated suppression. One of the simplest explanations for our failure to achieve significant suppression with solid phase coupled stimuli is that the target of Treg-mediated suppression *in vitro* is not the responder T cell but the APC. A wide variety of cell types have been described as direct targets of Treg-mediated suppression (Table 1), yet after 20 years of study, it remains unclear whether the APC or the responder T cell or both are targeted by Tregs in the widely used *in vitro* suppression assay. While multiple mechanisms of Treg-mediated suppression have been proposed (see below), suppression of APC function or delivery of APC-derived co-stimulatory signals have achieved the greatest attention. If the APC is the primary target for Treg suppression *in vivo*, it would be ideal to employ an *in vitro* assay that would mimic the *in vivo* action of Treg.

**Table 1:** Cellular targets for Foxp3^+^ T regulatory-mediated suppression.

CD4^+^, CD8^+^ T cells
Dendritic cells
B cells
Macrophages
Osteoblasts
Mast cells
NK cells
NK T cells
Adipocytes
Endothelial cells
Fibroblasts
Muscle
Hair follicle stem cells

## Treg Defects in Autoimmune Disease

Why is it important to have a reliable *in vitro* assay for Treg suppressor function? It has been proposed and in fact widely accepted that defects in Treg function play an important role in the pathogenesis of autoimmune disease in man (5). While some early studies claimed that patients with certain autoimmune diseases had a decreased percentage or even absolute number of Treg in their peripheral blood, the overwhelming consensus today is that patients with autoimmune diseases have normal numbers of Treg at least in their circulation. A defect in numbers in target organs remains possible, but difficult to assess in man. It therefore follows that Tregs from patients with autoimmune diseases must be functionally abnormal. The number of autoimmune diseases with purported defects in Treg function as detected *in vitro* has recently been summarized by Grant et al. (6). Defects in virtually all the common autoimmune diseases including SLE, MS, T1D, RA, autoimmune thyroid disease, psoriasis, IBD, primary biliary sclerosis, autoimmune hepatitis, and primary sclerosing cholangitis have been described. Indeed, it would be difficult to publish a paper claiming normal Treg function in any of these diseases. There are a number of reasons for defective Treg suppression *in vitro* in autoimmune disease:

1. Environmental—the production of pro-inflammatory cytokines by APC such as IL-6 (7) which can provide a potent co-stimulatory signal for T effector cell expansion and render the responder T cells resistance to suppression. IL-6 could also act on Treg cells and reverse their suppressive function or result in their conversion to Th17 cells.
2. T effector cell intrinsic resistance to suppression.
3. Treg intrinsic defects including defective generation, survival, stability, or altered TCR repertoire. Finally, specific defects in one of the proposed mechanisms of Treg-mediated suppression.

While dissection of which of these factors are operative in a given autoimmune disease is clearly doable in a well-characterized animal model, in human disease in the presence of normal numbers or percentages of Treg cells, one must rely on *in vitro* assays of suppressor function. The question to be addressed is whether *in vitro* suppression assays are capable of detecting major or even minor alterations in Treg function that mimic their defective function *in vivo*. The approach I have used to begin to address this question is to ask whether defects in Treg suppression *in vitro* can be detected with Treg cells derived from mice who develop autoimmune disease secondary to a deletion or mutation of a given gene specifically in Treg cells [Traf3 (8), CD28 (9), id2/id3 (10), ubc13 (11), Itch (12), NF-κB p65 (13), Helios (14), ThPoK/LRF (15), A384Tmutant of Foxp3 (16), and EZH2 (17)]. The thymic development of Treg is normal in all these strains and all have normal numbers of Treg cells; while all have moderate to severe autoimmune disease, but all have normal Treg suppressor function *in vitro*. Notably, when tested, Treg from many of these strains exhibit abnormal function *in vivo* in their capacity to suppress the adoptive transfer of IBD in immunodeficient mice following the transfer of naïve T cells. In a number of other studies of mouse strains with selective deletion of genes in Treg cells and resultant manifestation of severe autoimmunity [Bach2 (18), satb1 (19), IRF-4 and Blimp1 (20), and LKB1 (21)], the investigators have not even bothered to test Treg suppressor function *in vitro*.

**Figure 1:**
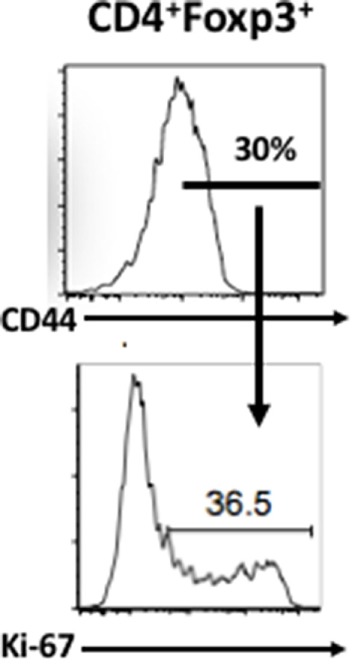
T regulatory Treg cells represent one of the most active cycling lymphocyte populations *in vivo*. After gating on Foxp3^+^ T cells, we then gated on the activated/effector/memory subset as define by high levels of CD44 expression. The CD44^hi^ population was then stained for Ki-67 expression. Ki-67 positivity reflects cell division over the previous 48-h period.

What factors could account for the failure of *in vitro* suppression assays to detect defects in Treg suppressor function? The Foxp3^+^ Treg population is heterogeneous and can be broadly subdivided into a naïve/quiescent/resting cell subpopulation and into a memory/effector/activated subpopulation. These two populations in the mouse can be distinguished by the differential expression of CD44 (22) or Ly-6C (23). The memory/effector subpopulation (CD44^hi^, Ly-6C^−^) appears to undergo increased TCR signals *in vivo* based on increased levels of CD5 expression and CD3ζ phosphorylation (23). Most importantly, the memory population contains a high percentage of cycling cells (~10%/day) based on Ki-67 staining (Figure 1). By contrast, when analyzed *in vitro*, Treg are characterized as anergic or non-responsive and fail to proliferate when stimulated with anti-CD3 alone, when stimulated with combinations or anti-CD3 and anti-CD28, or with high concentrations of IL-2 (2). The memory phenotype subpopulation manifests much higher suppressive activity *in vivo* (23). Furthermore, deletion of TCR expression from Treg results in a selective loss of the cycling MP Treg combined with a loss of Treg-mediated suppressor function *in vivo* (24). Thus, one major distinction between Treg function *in vitro* versus *in vivo* is the failure to see proliferating Treg under any culture conditions *in vitro*. It is quite possible that the activated/memory/effector Treg do not survive *in vitro* and their function is, therefore, never actually measured in standard *in vitro* assays. As the proliferating memory phenotype Treg are the major suppressive population *in vivo*, the relationship of what we observe in suppression assays *in vitro* to their physiologic suppressive function vivo remains unclear. While this conclusion is primarily based on studies with mouse Treg cells and human Treg cells may manifest different properties, I remain skeptical that we can use *in vitro* assays to define a defect in Treg suppressor function in autoimmune disease in man.

## Mechanisms of Treg-Mediated Suppression

**Figure 2:**
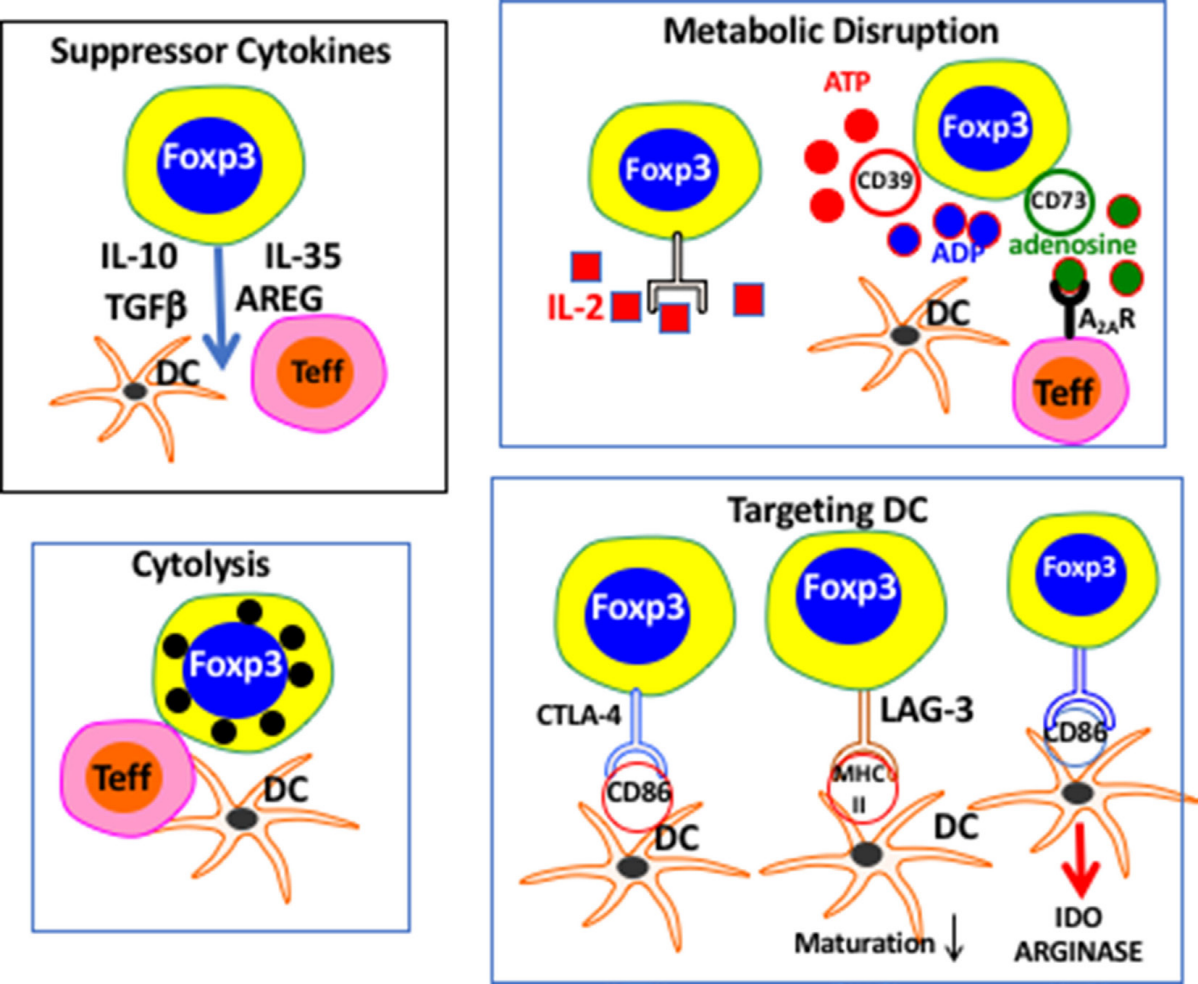
Proposed pathways of T regulatory (Treg)-mediated suppression. The pathways are roughly divided into different mechanistic categories. It remains unclear which or how many mechanisms are used by Treg under physiologic conditions *in vivo*.

One of the fundamental questions that one can raise regarding defects in Treg function is which mechanism of Treg-mediated suppression is actually defective? I have summarized (Figure 2) many of the proposed pathways by which Treg may manifest their suppressor effector function including release of soluble suppressor factors, cytolysis, disruption of metabolic pathways, and pathways used to selectively target DCs. The prevailing view in the field is that there is not one universal pathway by which Treg mediate suppression and that Treg have the luxury of picking from this large list of mechanisms to find one (or more) suitable for a particular situation or inflammatory niche. In fact, there are very few *in vivo* studies clearly supporting this hypothesis. One common mistake is that neutralization of a given pathway, for example, blocking the action of IL-10 (25) or TGF-beta with resultant loss of suppression, indicates that Treg are using only that pathway to mediate suppression. The alternative explanation is that the contribution of these suppressor cytokines is necessary, but not sufficient, for Treg-mediated suppression. Thus, in the xeno-graft versus host disease (GVHD) model (26) production of TGF-beta by Treg is required for prevention of disease, but Treg could also using other pathways at the same time. Indeed, Treg production of TGF-beta may only be required under “super-inflammatory” conditions such as xeno-GVHD, as mice with a selective deletion of TGF-beta in Treg do not exhibit an autoimmune phenotype (27). A similar scenario can be proposed for the requirement of IL-10 production for Treg-mediated protection from IBD, but not for the much less inflammatory autoimmune gastritis where IL-10-deficient Treg are fully protective (28).

The leading candidate for the most predominant suppressor mechanisms utilized by Treg is the downregulation of the expression of CD80/CD86 expression on DCs which is mediated by CTLA-4 expressed on Treg cells. It was first noted that Treg were the only lymphocyte population that expressed CTLA-4 constitutively and several early studies demonstrated that Treg suppression could be reversed *in vitro* (29) and *in vivo* (30) by anti-CTLA-4. This model received strong support for the studies of Wing et al. (31) which demonstrated that selective deletion of CTLA-4 expression from Treg resulted in the rapid development of autoimmune disease. Furthermore, Qureshi et al. (32) demonstrated that CTLA-4 was capable of selectively removing CD80/CD86 from the cell surface of DCs by a process of transendocytosis ultimately resulting in the degradation of CD80/CD86 within the Treg. Taken together these studies appear to offer a solid experimental foundation that this pathway is the major one utilized by Treg. However, several more recent studies suggest that the function of CTLA-4 in Treg is considerably more complex. First, it should be pointed out that in the studies of Qureshi et al. (32), CTLA-4 on activated conventional T cells could also mediate the transendocytosis of CD80/CD86. Thus, this pathway is not specific for Treg. Second, the recent studies of Paterson et al. (33) which demonstrated that specific deletion of CTLA-4 from the adult mouse Treg resulted in enhanced Treg proliferation *in vivo* and was accompanied by increased Treg suppressor function *in vivo*. Similarly, we have observed (34) that the homeostatic proliferation of Treg *in vivo* can be markedly enhanced by treatment of mice with anti-CTLA-4. The enhanced proliferation of Treg in this model was accompanied by enhanced proliferation of memory phenotype CD4^+^ and CD8^+^ T cells consistent with a loss of Treg suppressor function. Thus, after almost 20 years of intensive study, the role of CTLA-4 in Treg function remains unclear.

The second pathway of Treg-mediated suppression that deserves further discussion is whether consumption of IL-2 by Treg plays any role in Treg-mediated suppression. When we first presented the results of our Treg suppression assays in one of our joint lab meetings some 20 years ago, Bill’s first reaction was that they must be inhibiting by functioning as “IL-2 sinks” a concept originally proposed in the early 1980s (35). We always took Bill’s advice seriously and were then obligated to rule out this mechanism. We demonstrated that Treg inhibited proliferation by blocking the induction of IL-2 mRNA production in the responder T cell (2) and this observation was confirmed by many groups (36). The one exception being the studies of Pandiyan et al. (37) who claimed that Treg consume IL-2 and inhibit the proliferation of Foxp3^−^ T cells leading to Bim-mediated apoptosis. A number of observations have biased me against the concept of the “IL-2 sink” as an important pathway of Treg-mediated suppression: (A) It is widely assumed that because Treg express high levels of CD25 that they have high number of high affinity IL-2 receptors. In fact, no one has determined the number of high-affinity IL-2 receptors on Treg and it is likely that while they probably express in the range of 50,000 CD25 molecules that they express at least a log lower CD122 and CD132 molecules resulting in a level of expression of the high affinity IL-2R (the tri-molecular complex) similar to that seen on activated Foxp3^−^ CD4^+^ T cells. (B) The addition of exogenous IL-2 has no effect on Treg-mediated suppression of IL-2 production by CD4^+^ Foxp3^−^ T cells at the mRNA level (38). (C) In a trans-species model where human Treg can efficiently suppress mouse responder cells, the addition of a blocking anti-human CD25 had no effect on the suppressive function of the human Treg (4). (D) While IL-2 is critical for T cell proliferation and expansion *in vitro*, the expansion of CD4^+^Foxp3^−^ T cells *in vivo* in response to antigen stimulation occurs in the absence of IL-2 signaling, as antigen-specific T cells lacking CD25 expression expand as well as wild-type T cells following antigen recognition (28).

In addition to potentially functioning as an “IL-2 sink” for the inhibition of T effector proliferation, IL-2 may also play a critical role to support the maintenance of Foxp3 expression, Treg survival, and Treg proliferation by triggering the STAT5 pathway. However, it should be noted that the Treg subpopulation that appears to be responding to IL-2 homeostatically is the resting Treg population, not the activated cycling population suppressive population. In our studies, IL-2 played no role in Treg cycling *in vivo* (22). Chinen et al. (39) have attempted to resolve some of these issues by deleting expression of CD25 from Treg in combination with the expression of a constitutively active form of STAT5. The expression of the active form of STAT5 rescued mice from the autoimmune disease present in the CD25 deficient mice. These studies revealed that expression of CD25 on Treg was not needed for suppression of CD4^+^ responder T cells, but IL-2 consumption by CD25 expressed on Treg played a major role in suppression of CD8^+^ T cells. One explanation for this dichotomy is that CD8^+^ T cells are more sensitive to IL-2 signaling than CD4^+^ T cells. While these elegant genetic studies appeared to resolve the issue of IL-2 consumption at least for suppression of CD4^+^ T cell responses, more recent studies have shown that Treg cells expressing phospho-STAT5 localize in clusters in lymph nodes with IL-2 producing CD4^+^ Foxp3^−^ T cells (40). This localized response of Treg to IL-2 signaling also appeared to enhance their suppressive function. Thus, while deprivation of CD4^+^ effector T cells of IL-2 by Treg may not be play a role in suppression, the action of IL-2 locally produced by T effectors on Treg may be critical for their optimal suppressive activity presumably mediated by pathways other than IL-2 consumption. Indeed, we demonstrated over a decade ago that the initial production of IL-2 by responder T cells was required to activate the suppressor function of Treg which in turn suppressed the subsequent production of IL-2 by responder T cells (38).

## Antigen-Specific Suppression Versus Polyclonal Suppression *In Vivo* and *In Vitro*

One of the major conclusions drawn from studies of Treg suppressor function *in vitro* using both polyclonal Treg cells and antigen-specific Treg cells is that following stimulation *via* their TCR, the suppressor effector function of Treg is completely antigen non-specific. Thus, once activated by their cognate antigen, Treg specific for antigen A could suppress the proliferation of T effectors specific for antigen B (41). This concept is supported by studies which demonstrated that antigen-specific Treg cells are more potent inhibitors of disease than polyclonal Treg (42). However, our understanding of the mechanisms of Treg-mediated suppression *in vivo* is in a less advanced stage that our understanding of Treg-mediated suppression *in vitro*. A number of fundamental questions need to be addressed including: (1) the site of suppression (target organ or lymphoid tissues), (2) do Tregs inhibit homing of effector cells to the target organ, (3) can polyclonal Treg migrate to the target organ, (4) does suppression *in vivo* require the continuous presence of the Treg, (5) is suppression reversible, or (6) has a permanent state of tolerance been induced. None of these questions has definitively been answered and solutions are needed for the development of rational Treg therapies. Most importantly, we need reductionist models *in vivo* that will allow each aspect of the activation of T effector cell response to be analyzed. The field appears to be satisfied with studies demonstrating defective Treg suppressive activity in the classic cell transfer model of induction of IBD using polyclonal Treg, as originally described by Powrie and collaborators (43). However, this model is very complex as disease may be mediated by different T effector subsets (Th1 or Th17), involves both anti-self and anti-non-self responses as contribution of the intestinal microbiome is critical. Very few studies have addressed how Treg with defects in transcription factor function or signaling pathways actually fail to mediate suppression *in vivo*.

## Reversal of Treg-Mediated Suppression

I have already discussed the significance of neutralizing Treg suppression with antibodies to suppressor cytokines. An extension of this approach to dissecting mechanisms of Treg-mediated suppression has been to reverse suppression with antibodies to cell surface antigens expressed on Treg cells that play a role in the process of suppression. We (44) and others (45) first described that polyclonal or monoclonal antibodies to a member of tumor necrosis receptor superfamily, the GITR (TNFRSF18), could reverse Treg-mediated suppression *in vitro*. However, this conclusion was rapidly drawn into question as CD4^+^ Foxp3^−^ T cells can also express the GITR and more importantly expression of the GITR is rapidly upregulated on Foxp3^−^ T cells following TCR activation. Indeed, when we cultured combinations of WT and GITR^−/−^ Treg and effector T cells, we only observed reversal of suppression when the GITR was expressed on the responder T effector cells (46). Thus, engagement of the GITR on T effector cells by an agonistic antibody rendered the responder T cells resistant to suppression. It is highly likely that a similar induction of resistance to suppression in T effector cells is responsible for the purported reversal of Treg suppressor function (47) by agonistic antibodies to OX40 (CD137).

The most prominent member of the TNFRSF family that has been implicated in Treg function is TNF itself. Several studies with human T cells have reported that TNF could inhibit the function of Treg and that anti-TNF treatment of patients with RA resulted in restoration of defective Treg function when measured *in vitro* (48). However, TNF has also been demonstrated to have potent co-stimulatory function on T effector cells and it is likely that the TNF may have exerted its function on the T effectors rendering them resistant to suppression in a manner similar to the studies in the mouse with anti-GITR. Recent studies have failed to reproduce the deleterious effects of TNF on Treg function and have actually demonstrated that exposure of human Treg to TNF increased their expression of CD25 and Foxp3 (49).

It remains possible that future studies may identify cell surface antigens on Treg that are involved in Treg-mediated suppression. Hopefully, such studies will result in the development of agonistic antibodies that can either selectively expand Treg, enhance or alternatively reverse their suppressive function. While the studies discussed above were based on the enhanced expression of several members of this family on Treg (GITR, OX40, and TNFRII), the effects of these reagents *in vitro* and probably *in vivo* were mediated by their action as co-stimulatory molecules for T effector cells. Although this is a valuable lesson to have learned, it also has potentially clinical applications. In animal models, antibodies to the GITR have been shown to partially deplete Treg *in vivo* in the tumor microenvironment and to simultaneously provide co-stimulatory signals to CD4^+^ and CD8^+^ T effector cells resulting in inhibition of tumor growth (50). The usefulness of such reagents in the clinic remains to be evaluated.

## tTreg, pTreg, iTreg, and ex-Tregs

The concept that Treg cells could only be generated in the thymus was challenged by studies in the mid-2000s (51, 52) which demonstrated that Treg cells could be generated both *in vivo* (pTreg) and *in vitro* (iTreg) from peripheral CD4^+^ Foxp3^−^ T cells. TGF-beta plays a prominent role in the process, particularly *in vitro*. While there is little dispute about both of these phenomena, the significance, size, and function of the pTreg pool remains to be fully characterized. A significant impediment to progress has been a lack of a defined marker for thymus derived (tTreg). We have suggested that Helios is a useful marker of tTreg (53). Other groups have suggested that neuropilin-1 (Nrp1) is a more useful and more specific marker (54). There are important differences in the expression of these two antigens. First, Helios is a transcription factor thereby limiting its usefulness for isolation, although we now have generated a faithful Helios reporter mouse. Helios is expressed by 70–80% of Treg in peripheral lymphoid tissues and by a somewhat lower percentage (50–60%) of mucosal derived Treg. By contrast, Nrp1 is expressed by 85% of peripheral Treg. The mAb generated against mouse Helios cross-reacts with human Helios and reacts with 80% of Treg in human peripheral blood. Both Helios and Nrp1 can be expressed by conventional T cells in the mouse, although we have not been able to detect Helios expression in human non-Treg under any conditions *in vivo* or *in vitro* (55). The expression of Nrp1 by human Treg is unclear. One major deficiency of using Nrp1 as a marker of tTreg is that its expression is regulated by TGF-beta. Thus, pTreg generated in the central nervous system were shown to be suppressive, but uniformly expressed Nrp1; iTreg generated in culture in the presence of TGF-beta are uniformly Nrp1^+^ (54). Furthermore, the percentage of Nrp1^+^ Treg is greatly reduced in mice with a T cell-specific deletion of TGF-beta clearly demonstrating that the constitutive expression of Nrp1 is closely regulated by TGF-beta (unpublished observations).

For these reasons, we strongly favor the use of Helios as a definitive marker of tTreg. However, it is incumbent upon us to prove that this is the case. In order to study the differences between Helios^+^ and Helios^−^ Treg, we have generated a Helios-GFP/Foxp3-RFP double reporter mouse. The Helios^+^ Treg population expressed a more activated phenotype and had slightly higher suppressive capability *in vitro*. Both subsets were equivalent in their ability to suppress IBD *in vivo* and both subsets expressed a highly demethylated TSDR, with slightly higher demethylation in the Helios^+^ Treg subset. This result is consistent with the concept that pTreg generated *in vivo* are relatively stable (56). Upon transfer to normal mice, both Helios^+^ and Helios^−^ Treg cells maintained equal Foxp3 stability and Foxp3^+^Helios^+^ Treg maintained stable expression of Helios. Preliminary analysis of the TCR repertoire of both subsets by deep sequencing revealed little to no overlap of the two populations consistent with distinct origins of the subsets (unpublished observations). Taken together, our data indicate that Helios expression can differentiate two distinct populations of Treg with overlapping functions, most likely representing tTreg (Helios^+^) and stable peripherally induced pTreg (Helios^−^). However, considerable controversy still exists regarding the use of Helios as a marker for tTreg (57, 58) and caution should still be exerted when using this marker.

Several studies over the past 5 years have challenged the notion that Foxp3^+^ Treg cell lineage is stable and have raised the possibility that Treg cells can lose Foxp3 expression particularly when present in an inflammatory milieu resulting in “reprogramming” of Treg to potentially pathogenic T effector cells (59). As Treg express an anti-self biased TCR repertoire, these re-programmed Treg would represent a potential potent population of T cells capable of inducing autoimmune disease. As complete deletion of Treg from adult mice results in exuberant inflammation and death in 10–15 days (60), the maintenance of Treg stability is critical to the survival of the host. For this reason, we favor the view that most tTreg are very stable and are unlikely to lose Foxp3 expression. Nevertheless, the studies of Treg instability are convincing and need to be addressed. One possibility is that the unstable population of Treg primarily develops from the pTreg population. pTregs represent logical candidates for instability even though most may have a demethylated TSDR. Alternatively, a minor population of pTreg may not be fully committed to the Treg lineage. The studies of Miyao et al. (61) clearly demonstrate the existence of a small population of Treg that can readily lose Foxp3 expression and can rapidly expand *in vivo* and thus appear to represent a large percentage of Treg in fate mapping studies. Other studies suggest that tTreg can also manifest Foxp3 instability (62). The recent demonstration (63) of a population of unstable and dysfunctional Treg in the tumor microenvironment that still maintain Foxp3 expression adds further complexity to our understanding of the role of “ex-Tregs.” Studies in the future need to resolve the issue of tTreg versus pTreg and the role of Treg stability. It is unclear if the loss of Treg stability contributes to the pathogenesis of any human autoimmune diseases, but this is a difficult issue to address experimentally.

## The Future

Although many of the issues posed above have not yet been completely addressed, the use of Treg for cellular biotherapy has already reached the clinic in studies for the prevention of GVHD following stem cell transplantation (64) as well as autoimmune disease (65). The successful use of low-dose IL-2 treatment to expand Treg in two clinical trials (66, 67) has stimulated great interest. A recent perusal of ClinicalTrials.gov has revealed 181 proposed studies involving the use of Treg cells and a number of trials of low dose IL-2 treatment alone or in combination with Treg cellular therapy are planned. Of note, no studies are listed using specific pharmacologic manipulation of Treg function or using monoclonal antibodies to enhance or suppress Treg function. My own view is that the development of such reagents is required before we will have the necessary tools for the therapeutic manipulation of Treg cell function in man. As emphasized in this review, further studies of the biological properties of Treg, particularly the specific mechanisms of suppression utilized in given disease states, are needed as the foundation for the development of pharmacologic reagents.

In conclusion, I would like to thank Bill Paul for supporting my career for the past 40 years. He was always available for discussions and freely provided advice on an almost daily basis. I will miss him most during our weekly data clubs and journal clubs. He was a master at pointing out great science and terrific critique of marginal experiments.

## Author Contributions

The author confirms being the sole contributor of this work and approved it for publication.

## Conflict of Interest Statement

The author declares that the research was conducted in the absence of any commercial or financial relationships that could be construed as a potential conflict of interest. The handling Editor declared a shared affiliation, though no other collaboration, with the author.
